# Combined treatment with enteric neural stem cells and chondroitinase ABC reduces spinal cord lesion pathology

**DOI:** 10.1186/s13287-020-02031-9

**Published:** 2021-01-06

**Authors:** Benjamin Jevans, Nicholas D. James, Emily Burnside, Conor J. McCann, Nikhil Thapar, Elizabeth J. Bradbury, Alan J. Burns

**Affiliations:** 1 Stem Cells and Regenerative Medicine, UCL Great Ormond Street Institute of Child Health, London, UK; 2 Present Address: German Centre for Neurodegenerative diseases (DZNE), Bonn, Germany; 3 Regeneration Group, The Wolfson Centre for Age-Related Diseases, Institute of Psychiatry, Psychology & Neuroscience, King’s College London, Guy’s Campus, London, UK; 4 Neurogastroenterology and Motility Unit, Department of Gastroenterology, Great Ormond Street Hospital, London, UK; 5 Present Address: Department of Paediatric Gastroenterology, Hepatology and Liver Transplant, Queensland Children’s Hospital, Brisbane, Australia; 6 Department of Clinical Genetics, Erasmus Medical Center, Rotterdam, The Netherlands; 7 Present Address: Gastrointestinal Drug Discovery Unit, Takeda Pharmaceuticals International, Cambridge, USA

**Keywords:** Spinal cord injury, Stem cells, Enteric neural stem cells, ChABC

## Abstract

**Background:**

Spinal cord injury (SCI) presents a significant challenge for the field of neurotherapeutics. Stem cells have shown promise in replenishing the cells lost to the injury process, but the release of axon growth-inhibitory molecules such as chondroitin sulfate proteoglycans (CSPGs) by activated cells within the injury site hinders the integration of transplanted cells. We hypothesised that simultaneous application of enteric neural stem cells (ENSCs) isolated from the gastrointestinal tract, with a lentivirus (LV) containing the enzyme chondroitinase ABC (ChABC), would enhance the regenerative potential of ENSCs after transplantation into the injured spinal cord.

**Methods:**

ENSCs were harvested from the GI tract of p7 rats, expanded in vitro and characterised. Adult rats bearing a contusion injury were randomly assigned to one of four groups: no treatment, LV-ChABC injection only, ENSC transplantation only or ENSC transplantation+LV-ChABC injection. After 16 weeks, rats were sacrificed and the harvested spinal cords examined for evidence of repair.

**Results:**

ENSC cultures contained a variety of neuronal subtypes suitable for replenishing cells lost through SCI. Following injury, transplanted ENSC-derived cells survived and ChABC successfully degraded CSPGs. We observed significant reductions in the injured tissue and cavity area, with the greatest improvements seen in the combined treatment group. ENSC-derived cells extended projections across the injury site into both the rostral and caudal host spinal cord, and ENSC transplantation significantly increased the number of cells extending axons across the injury site. Furthermore, the combined treatment resulted in a modest, but significant functional improvement by week 16, and we found no evidence of the spread of transplanted cells to ectopic locations or formation of tumours.

**Conclusions:**

Regenerative effects of a combined treatment with ENSCs and ChABC surpassed either treatment alone, highlighting the importance of further research into combinatorial therapies for SCI. Our work provides evidence that stem cells taken from the adult gastrointestinal tract, an easily accessible source for autologous transplantation, could be strongly considered for the repair of central nervous system disorders.

## Background

Spinal cord injury (SCI) is a devastating condition with an estimated UK prevalence of approximately 40,000 [1]. The injury process occurs biphasically, beginning with axonal damage and cell death as a direct result of the initiating trauma [2], and proceeding with haemorrhage [3], immune infiltration [4], extensive cell death/axonal degeneration and astroglial activation [5]. Activated astrocytes and other reactive cells secrete chondroitin-sulfate proteoglycans (CSPGs) [6–11], which induce axon growth cone collapse and prevent axons from sprouting across the injury site [12, 13]. Currently, there is no effective cure for SCI.

Cell-based therapies for SCI hold great promise in terms of cell replacement [14, 15], modification of the inhibitory micro-environment [16] and endogenous neuroprotection [17]. Stem cells from various sources have been examined. Mesenchymal stem cells have been shown to nullify the inhibitory micro-environment that develops following SCI, resulting in increased endogenous regeneration [18]. Transplantations of olfactory ensheathing glia (OEG) isolated from the olfactory bulb have resulted in considerable motor and sensory improvements after injury [19]. Neural stem cells taken from the subventricular/subgranular zones of the brain, or the ependymal canal of the spinal cord, are able to replace the cells lost due to the injury process and have resulted in significant improvements [20]. Embryonic stem cells have the ability to form all required cell types [21], although their use raises some ethical concerns [22]. Finally, induced pluripotent stem cells (iPSCs) are advantageous in many ways, including their extensive reprogramming potential and the possibility of autologous transplantations [23]. All of these stem cell sources show exciting promise [24–29], but no single stem cell source represents an ideal solution. Clearly, therefore, potential alternative sources should be evaluated to determine whether they represent a significant advantage over existing options.

Enteric neural stem cells (ENSCs) harvested from the enteric nervous system (ENS), the intrinsic innervation of the gastrointestinal (GI) tract, are an attractive option. ENSCs persist into late adulthood [30, 31] and can be harvested via routine GI procedures such as endoscopy, providing the potential for autologous transplantation. The therapeutic utility of ENSCs for enteric disorders has been previously explored [32, 33], most recently following transplantation into a murine enteric neuropathy model, where ENSCs successfully engrafted into the host tissue and restored gut function [34]. Interestingly, similarities between the central nervous system (CNS) and ENS has led to several studies evaluating the potential of ENSCs for CNS disorders [35–37]. Indeed, our lab has previously utilised an embryonic chick model to examine the potential of ENSCs for SCI repair [38]. We found that chick ENSCs expressed markers of cell types relevant for SCI repair, suggesting that ENSCs could produce the required cell types without genetic reprogramming, reducing the risk of genomic instability and associated tumorigenic potential of some other stem cell sources [39, 40]. We also reported the ability of ENSCs to survive transplantation into, and form bridging structures across, the injury zone. However, the embryonic chick model of SCI presents a milder injury zone than that which develops following adult mammalian SCI. Therefore, for progression to a mammalian SCI model, we sought to enhance the ability of transplanted ENSCs to engraft into host tissue. Several laboratories have shown the ability of the bacterial enzyme chondroitinase ABC (ChABC) to digest the inhibitory CSPGs within the glial scar, leading to increased endogenous sprouting and recovery of motor function [41–45]. We hypothesised that combined treatment of SCI with ENSC transplantation and ChABC application would prove more effective than either therapy alone, as has been shown in previous publications examining the potential of alternative neural stem cell sources [46, 47].

Utilising a rat contusion model of SCI, we demonstrate for the first time long-term survival of ENSCs within the spinal cord injury zone, with ENSC grafts evident at 16 weeks post-transplantation. The combined treatments resulted in a reduction of the cavity area, and retrograde tracing studies revealed both that ENSC-derived projections extended through and past the injury zone and that ENSC transplantation resulted in an increase in the number of cells projecting caudally through the injury site. Further, at 16 weeks post-transplantation, the combined treatment resulted in a modest, but significant functional improvement, and we provide preliminary safety evidence for a lack of ectopic ENSC migration from the transplant site over a period of 16 weeks. These results strongly encourage further investigation into the use of ENSCs for a range of CNS disorders.

## Methods

### Animals

SCI was induced in 26 adult female Sprague-Dawley rats (180–200 g; Harlan Laboratories). Two animals died during surgery. Animals were housed together under standard husbandry conditions with a 12-h light/dark cycle and access to food and water ad libitum. All experimental procedures were carried out in accordance with the UK Animals (Scientific Procedures) Act 1986 under Home Office Project Licence 70/8032. Following SCI, animals were randomly assigned to one of four groups (no treatment; LV (lentivirus)-ChABC injection only, ENSC transplantation only and ENSC transplantation+LV-ChABC injection; *n* = 6 per group) by an experimenter whom, following the surgical procedures, played no further part in the investigation. Animals within the no treatment group received sham injections of 0.9% sterile saline, and animals in the single treatment groups (ENSC transplantation only and ChABC only) received additional sham injections of 0.9% saline so that all groups received the same number of injections. All analyses were conducted while blinded to the treatment groups.

### Cell isolation, culture and enrichment

The intestines of 3 female neonatal Sprague-Dawley rat pups (P7) were harvested into Mg^2+^/Ca^2+^ free phosphate-buffered saline (PBS, 0.1 mol L^− 1^, pH 7.2) under sterile conditions and the mucosal layer removed via fine dissection. Intestinal cells from the *muscularis* were dissociated and plated (~ 50,000 cells mL^− 1^), onto 2% fibronectin-coated cell culture dishes. Cells from individual animals were cultured separately to allow the quality control of harvests. Two days after tissue harvest, cells were collected for FACS analysis. Following labeling with anti-p75 FITC-conjugated primary antibody (Eurogentec, Belgium), cells were sorted using a MoFloXDP cell sorter (Beckman Coulter, UK), 530/40 filter set and re-plated at the original density. Media was refreshed every 2–3 days (DMEM F12 (Sigma Aldrich, UK), N2 (Gibco Life Technologies, UK), B27 (Gibco Life Technologies, UK), primocin (InvivoGen, UK), FGF and EGF (20 ng mL^− 1^, Peprotech, UK). Prior to transplantation, cells were labelled with a self-inactivating (SIN) second-generation HIV-1-based lentivirus containing mutated Woodchuck Posttranscriptional Regulatory Element (WPRE) downstream of enhanced green fluorescent protein (eGFP) [48] to allow for post-transplantation visualisation. For transplantation studies, neurospheres from all animals were collected and pooled, dissociated into a single-cell suspension, concentrated by centrifugation (1000 rpm for 5 min) and counted using a haemocytometer with Alcian Blue.

### Contusion injury surgery

**Fig. 1:**
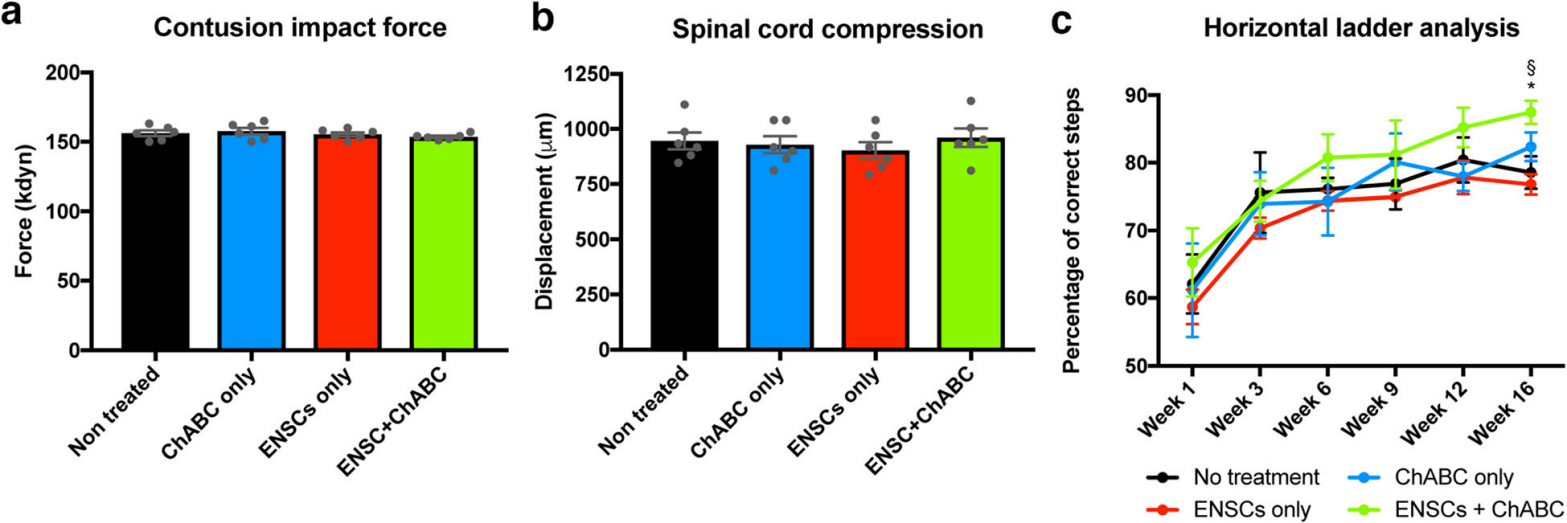
Spinal cord contusion injuries resulted in difficulty traversing the horizontal ladder, which improved over the course of the study. Spinal cord injuries were induced with an Infinite Horizons impactor. Applied force (**a**) and spinal cord displacement (**b**) were consistent between animals. Following injury, the ability of each rat to traverse the horizontal ladder was assessed at regular time points (**c**). All rats improved over the course of the study. At the 16 week time point, there was a significant difference between the ENSCs+ChABC and the non-treated group (* indicates significance (*p* = < 0.05) and between the ENSCs+ChABC and the ENSC-only group (§ indicates significance (*p* = < 0.005). Data are represented as mean ± s.e.m

Contusion injury was induced as previously described [49]. Briefly, rats received perioperative analgesia (Carprofen 5 mg kg^− 1^) and were anaesthetized (ketamine, 60 mg kg^− 1^, and medetomidine, 0.25 mg kg^− 1^ administered i.p.), and a laminectomy performed at T10. Core temperature was maintained at 37 °C during surgery. A contusion injury of 150 kdyne was induced (Infinite Horizon Impactor, Precision Systems Instrumentation, Lexington, KY) as previously described [50]. Measurements of the administered force and spinal cord displacement were recorded to ensure consistency of injuries between animals (Fig. 1a, b). Following anaesthesia, reversal (atipamezole hydrochloride, 1 mg kg^− 1^ administered subcutaneously) animals received saline for rehydration (3–4 mL, once daily for 3 days), post-operative analgesia (Carprofen 5 mg kg^− 1^, once daily for 2 days) and antibiotics (Baytril, 5 mg kg^− 1^, 1 week course).

### Chondroitinase ABC lentiviral vector production

The lentiviral vector containing the Proteus vulgaris ChABC gene (LV-ChABC) was produced as previously described [50] and provided as a generous gift from Prof Joost Verhaagen (Supp. Figure 1). Briefly, the ChABC gene was modified via the removal of five cryptic n-glycosylation sites and resynthesized using mammalian-preferred codons [51]. This was subcloned into a lentiviral transfer vector to produce an integrating, self-inactivating vector, pseudotyped with VSV-G [52]. Viral particles were concentrated via ultracentrifugation and titration using a p24 antigen enzyme-linked immunosorbent assay (ELISA) assay to 1 × 10^9^gc (genome copies) mL^− 1^, suspended in 0.9% sterile saline solution.

### Cell transplantation and LV-ChABC injection

Rats receiving ENSC transplantations/LV-ChABC treatment were anaesthetised and the spinal cord re-exposed 3 days following contusion, as above. All injections were conducted with a pulled glass needle at a depth of 1 mm. Individual rats received 1 × 10^6^ cells, divided into 3 injections: 1 mm rostral to the injury epicentre, into the epicentre and 1 mm caudal to the epicentre, each of 0.5 μL, delivered at a rate of 200 nL min^− 1^ using an ultra micropump III (World Precision Instruments, Europe). Rats undergoing LV-ChABC treatments received two intraspinal injections immediately following ENSC transplantation, at the same rostral and caudal sites as the stem cell transplantations. 0.5 μL of LV-ChABC was delivered per site at a rate of 200 nL min^− 1^. Following all injections, the needle was left in place for 2 min before retraction. Anaesthesia was then reversed and animal recovery monitored as above. Previous work in our group has demonstrated no significant effect of control (GFP) lentiviral constructs on cavity formation, CSPG pathology, the density of fibres passing through the lesion or cell survival compared to sham injections of 0.9% saline [50] and so sham injections in the no treatment group consisted of saline only.

### Axon tracing

Retrograde tracing was used to determine whether transplanted ENSCs could project axons across the injury site to reach rostral regions, and to quantify the number of cells projecting axons caudally through the injury site. One week prior to study completion, 3 rats (*n* = 3) from the non-treated, ENSC transplantation only and ENSC+ChABC groups were randomly selected. Animals were anaesthetised and a T12 laminectomy performed (caudal to the injury site and site of ENSC/ChABC injections, which were performed at T10). A pulled glass pipette connected to a Microdrive pump (NanoLiter 2010 Injector/Micro 4 Controller, World Precision Instruments) was lowered into the midline of the spinal cord to an initial depth of 300 μm, retracted 100 μm and injected at a rate of 200 nl min^− 1^ with 2 μl of Fluorogold (FG) (4%, dissolved in sterile saline, 0.9%). Following injection, the needle was left in place for 2 min and slowly withdrawn.

### Horizontal ladder training

The horizontal ladder test was used as a measure of locomotor ability/recovery. Prior to surgery, animals were trained on the task to provide baseline data. Starting 1 week after surgery, rats were assessed weekly until study completion by an examiner blinded to the treatment groups. Rats were placed individually onto a horizontal ladder 1 m in length, with irregularly placed rungs. Testing was captured using a Sony DCR-SX30E Handycam, and the total number of forelimb and hindlimb footslips was counted for 3 runs.

### Sacrifice and tissue harvest

16 weeks post-surgery, rats were deeply anaesthetized (sodium pentobarbital, Euthatal; 80 mg kg^− 1^, i.p.) and perfused through the ascending aorta with 0.9% saline, followed by ice-cold 4% PFA. The spinal cord was harvested at the level of T10 (+/− 5 mm), along with the left lung, right lateral lobe of the liver, right kidney and spleen. Samples were post-fixed at 4 °C overnight and stored in PBS. Samples for sectioning were cryoprotected overnight at 4 °C in 30% sucrose and transferred to OCT (Thermo Scientific, USA). Samples were orientated for sagittal sectioning, frozen using − 65 °C isopentane and stored at − 80 °C prior to sectioning. Frozen OCT-embedded samples were sectioned serially (20 μm) using a Leica Cryostat at − 22 °C and slides stored at − 20 °C. For all quantitative measurements, every fifth section was used.

### Immunostaining

Thawed cryosections or cell cultures were post-fixed with 4% PFA for 8 min and washed in 1XPBS. For immunofluorescent staining, samples were blocked with 1% bovine serum albumin (Sigma Aldrich, UK), 0.15% glycine (Fisher Scientific, UK), 0.1% Triton X-100 (Sigma Aldrich, UK) and in 1XPBS for 1 h and incubated in primary antibody (Table 1), diluted in blocking solution, overnight at 4 °C. Secondary antibody (Table 2) was applied in blocking solution for 2 h (RT). Coverslips were mounted using Vectashield (hard set with DAPI, Dako, UK). Slides were stored at 4 °C. For immunohistochemical staining, endogenous peroxidase was quenched using 3% H_2_O_2_ and 10% methanol in PBS. Non-specific binding was blocked with 5% normal goat serum in PBS containing 0.1% Triton X-100. Samples were incubated with primary antibody overnight at RT diluted in blocking solution. Biotinylated secondary antibody diluted in blocking solution was applied for 2 h at RT followed by incubation with avidin-biotin-peroxidase (ABC Elite kit, Vector Laboratories, Burlingame, CA, USA), and the colour reaction was developed using 3,3′-diaminobenzidine kit (Vector Laboratories). Sections were air-dried and coverslipped using Depex (Sigma-Aldrich, St. Louis, MO, USA).

**Table 1:** Primary antibodies

Protein target	Host species	Concentration	Supplier
TuJ1	Mouse	1:500	Covalence
GFP	Mouse	1:500	Invitrogen
GFP	Rabbit	1:500	Invitrogen
Ki67	Rabbit	1:500	Novocastra
nNOS	Rabbit	1:500	Invitrogen
5HT	Mouse	1:500	Millipore
Fluorogold	Rabbit	1:10,000	Fluorochrome

**Table 2:** Secondary antibodies

Target	Host species	Concentration	Supplier	Emission wavelength
Rabbit	Goat	1:500	Invitrogen	488
Rabbit	Goat	1:500	Invitrogen	568
Mouse	Goat	1:500	Invitrogen	568
Nuclei (DAPI)	N/A	1:1000	Sigma Aldrich	350
Rabbit	Goat	1:300	Vector Biolabs	Biotinylated

### Eriochrome cyanine staining and analysis

Eriochrome cyanine (EC) staining is commonly used to detect myelin and was used in this study to demarcate white and grey matter within spinal cord sections to allow quantification of cavitation and tissue damage as previously described [43]. Briefly, slides were thawed at RT, dehydrated in an ascending series of ethanol solutions and cleared in Histochoice (Sigma Aldrich, UK). Slides were rehydrated in a reverse series of ethanol solutions and immersed in EC solution (0.16% eriochrome Cyanine-R, 0.5% sulphuric acid, 0.4% iron chloride, in ddH_2_O). Slides were washed in ddH_2_O and differentiated in 0.5% aqueous ammonium hydroxide. Following a final ddH_2_O wash slides were dried at 50 °C and mounted using DPX (Merck Millipore, Germany).

Treatment groups were randomised prior to analysis. Sections were imaged using a Zeiss Axioplan microscope mounted with a Zeiss colour camera and analysed using Fiji (ImageJ) software [53]. If required, images were stitched using the MosaicJ plugin [54]. The spinal cord cavity area was quantified using the ‘magic wand’ tool set to a tolerance of 6. The injured tissue area + cavity area was quantified using the ‘magic wand’ tool set to a tolerance of 18, and the spinal cord cavity area value subtracted from this number to give the injured tissue area. In both instances, the area was quantified on every fifth section through the entire spinal cord. Except where noted, all analyses were conducted on at least 3 individual animals (*n* = 3) from each group, selected at random by an investigator blinded to the treatment groups.

### Cell survival/spread quantification

For all analyses, every fifth section was used. Serial spinal cord sections of ENSC-transplanted rats were assessed to determine both survival and spread of transplanted cells. Treatment groups were randomised prior to analysis. GFP antibody-labelled sections were imaged using a Zeiss Axioplan microscope mounted with a Zeiss colour camera and analysed using FIJI [53]. Images were stitched using the MosaicJ plugin [54]. For anterior/posterior and dorsal/ventral spread, the section with the greatest spread was chosen for each rat, and the extent of spread determined as the farthest GFP+ signal (cells or projections) in either direction. For left/right spread, the distance was calculated between the first and last serial sections in which GFP+ signal could be detected. The number of GFP+ cells was quantified using FIJI [53]. Background subtraction was utilised with a rolling ball of 10 pixels, followed by a median filter set to 1 pixel radius. Cell number was then determined by the ‘find maxima’ function, with a noise tolerance of 544. Quantification began at the first section to contain a positive GFP signal and proceeded sagitally until the last section containing GFP signal. The number of GFP+ cells in each section was pooled to give a total, summed cell count. The amount of GFP+ pixels was quantified using the ‘Threshold’ tool, set to a tolerance of between 44 and 255 (measurements limited to a threshold), and conducted on the same sections as the cell count analyses. Results were pooled to give a total, summed intensity.

### Quantification of Fluorogold+ cells

The number of Fluorogold+ cells rostral to the lesion was quantified using unbiased stereology. Every 5th section was used, with 5 sections examined per animal. The sections were selected to span the lesion epicentre. The entire spinal cord area rostral to the lesion was delineated under a 2× objective, and counting was performed under a 100× Plan-Apo oil objective, with a guard zone thickness of 1 μm at the top and bottom of each section. The optical fractionator probe (Stereo Investigator software version 9, MBF Biosciences, Williston, VT, USA) was used to count positive neurons, with a counting frame size of 60 × 60 and a sampling grid size of 268 × 268. Only cell bodies were counted, determined morphologically by a clearly defined cell border, the presence of a nucleus (clearly defined nuclear border with no positive staining inside) and at least one FG+ projection. The coefficient of error was calculated according to Gundersen and Jensen [55], with values < 0.1 accepted.

### PCR detection of transplanted cell spread

To determine whether ENSCs spread to ‘off-target’ regions following transplantation into the spinal cord, samples from peripheral organs (right medial lobe of the liver, spleen, right kidney and left lung) were analysed by PCR for the presence of *Gfp*. DNA was extracted from 10 samples (~ 30 mg) collected at random from each organ. Spinal cord cryosections of transplanted animals confirmed to contain transplanted GFP+ cells by fluorescent microscopy were used as a positive control. Samples were dried at RT, suspended in 25 μL proteinase K solution (1 μL proteinase K (Sigma Aldrich, UK) and 40 μL DNA extraction lysis buffer (100 mM Tris. Cl, 5 mM EDTA, 0.2%SDS, 200 mM NaCl, pH 8) and heated to 55 °C for 4 h. Digestion was halted by heating to 85 °C for 10 min. Cellular debris and other impurities were removed using salt precipitation. 1 μL from each of the 10 samples collected from each organ were pooled and diluted in 0.3 M NA-Acetate and 70% EtOH. Samples were incubated on ice for 30 min and centrifuged at 14,000 g for 30 min at 4 °C. The supernatant was discarded and the pellet washed in 70% EtOH. Samples were centrifuged at 14,000 g for 15 min at 4 °C, the supernatant discarded and the pellet re-suspended in 10 μL ddH_2_0. 1 μL of the precipitated DNA solution was used for each PCR reaction. PCR reactions were conducted in a PCT-200 Peltier Thermal Cycler (MJ Research Inc. Waltham, MA, USA) using HotStarTaq DNA Polymerase (Qiagen, Manchester, UK). Following amplification (Table 3) with primers designed for rat *Gfp* and *Gapdh* (Table 4), PCR products were analysed on a 2.5% agarose gel alongside a 25 bp hyperladder (Bioline, London, UK).

**Table 3:** PCR cycling programme

Step	Temp °C	Time
1	94	3 min
2	94	30 s
3	58	45 s
4; go to step 2, 35 cycles	72	30 s
5	72	2 min
6	4	Hold

**Table 4:** PCR primers

Probe target	Primer sequence	Product size	Tm
Gfp	F: CACATGAAGCAGCACGACTT R: TCCTTGAAGTCGATGCCCTT	167	59.13 59.02
Gapdh	F: GTTGTGGATCTGACATGCCG R: GGTGGAAGAATGGGAGTTGC	171	59.27 58.82
Sox10	F: ACCTCCACAATGCTGAGCTC R: CGCCGAGGTTGGTACTTGTA	160	60.04 59.76
Tuj1	F: TGACGAGCATGGCATAGACC R: AATAGGTGTCCAAAGGCCCC	192	59.9 59.66
Gls1	F: GTGTGTTCAAAGCAACATCGTT R: ACACCCCACAAATCAGGACT	195	58.57 58.85
nNos	F: AGGACAACGTTCCTGTGGTC R: CCGTCTCCCAGTTCTTGACC	150	59.89 60.04
Tph1	F: TGCGACATCAACCGAGAACA R: CGCAGAAGTCCAGGTCAGAA	172	59.97 59.68
Chat	F: TTTGATGGCATCGTCCTGGT R: CGAGATGGCCTTGGGTTTCT	166	59.67 60.04
Gad	F: GAGTCGTCTTGTGAGTGCCT R: GTTTGCTCCTCCCCGTTCTT	171	59.68 60.25
S100b	F: TCAGGGAGAGAGGGTGACAA R: TCATGACAGGCTGTGGTCAC	218	59.51 59.96

### qRT-PCR

Total RNA was extracted from p75+ cell cultures using an RNeasy Mini kit (Qiagen, Hilden, Germany), following the manufacturer’s instructions, and the total yield quantified using a NanoDrop 1000 (Thermo Scientific, UK). 100 ng RNA was used for first-strand cDNA amplification using SuperScript VILO cDNA Synthesis Kit (Life Technologies Ltd., Paisley, UK). Quantitative RT-PCR was performed using the ABI prism 7500 sequence detection system (Applied Biosystems) using the Quantitect SYBR Green PCR kit (Qiagen, Hilden, Germany) according to the manufacturer’s instructions. Reactions were performed in triplicate using region-specific primers for *Gapdh, Sox10, TuJ1, Gls1, Nos1, TPh1, Chat, Gad1* and *S100b* (Table 4). Gene expression levels were expressed relative to *Gapdh* (as a reference housekeeping gene), using a 1/∆Ct calculation.

### Statistical analysis

Data are expressed as mean ± s.e.m. GraphPad Prism software was used for all statistical analyses. Group comparisons of EC-demarcated lesion histology and comparisons of the number of FG+ cells rostral to the lesion were analysed using one-way ANOVA, followed by Tukey’s multiple comparisons post hoc test. Transplanted cell spread/survival was analysed using Student’s *t* test (two-tailed). *p* values of < 0.05 were taken as significant. Error bars represent standard error.

## Results

### Combined treatment with ENSCs+ChABC resulted in significant improvements in the horizontal ladder test only in week 16

Rats were assessed for their ability to traverse a horizontal ladder. In the early weeks following SCI, all rats struggled to correctly cross the ladder, with numerous ‘footslips’. Over the course of the study, all rats improved in this test (Fig. 1c). For the first 15 weeks, there was no significant difference between any of the groups in the percentage of correct footsteps. However, at week 16 (the latest time point examined), there was a significant difference between the ENSCs+ChABC and no-treatment group (78.5 ± 2.3 vs 87.47 ± 1.71, *p* = 0.0206) and between the ENSCs+ChABC and ENSC-only group (87.4 ± 1.71 vs 76.8 ± 1.51, *p* = 0.005).

### ENSCs cultured in vitro formed neurospheres containing dividing cells as well as mature neuronal subtypes

**Fig. 2:**
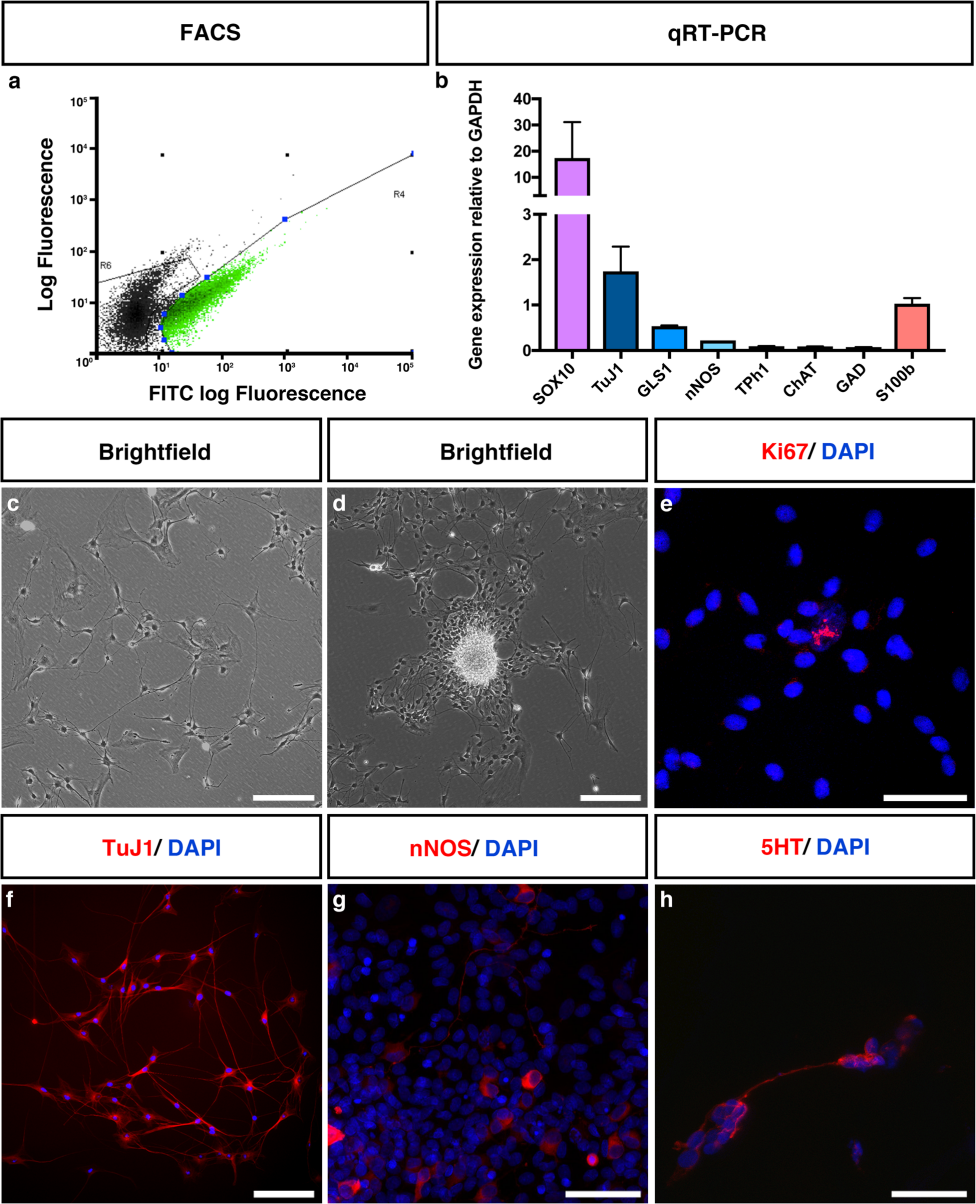
In vitro cultures of rat-derived ENSCs contain a heterogenous population of dividing progenitor cells and various neuronal subtypes. **a** Representative FACS plot showing isolation of p75 FITC-labelled ENSCs. Following FACS, 1 week-old p75+ cultures were analysed by qRT-PCR, revealing expression of Sox10 (neural crest cell progenitor cell marker), Tuj1 (pan-neuronal marker), Gls1 (glutamine), nNos (neuronal nitric oxide), Tph1 (serotonin), ChAT (acetylcholine), Gad (GABA) and S100b (glia) (**b**). **c**–**h** In vitro characterisation of ENSCs prior to transplantation. p75-sorted cell cultures displayed a characteristic neuronal morphology by 1 week in culture, including extension of fine interneuronal processes (**c**), and formed dense neurospheres by around 2 weeks (**d**). A small number of dividing cells were detected by Ki67+ staining (**e**). However, the vast majority of cells stained positive for the pan-neuronal marker TuJ1 (**f**), indicating neuronal differentiation. A subpopulation of cells stained positive for specific neuronal subtype markers, including nNOS (**g**) and 5HT (**h**). Data are represented as mean ± SEM. Scale bar—**c**, **d** 200 μm, **e** 50 μm, and **f**, **g**, **h** 100 μm

SCI results in extensive loss of a variety of neuronal subtypes from the injured tissue. To explore the potential of ENSCs to replace these lost cells, we first characterised them in vitro. ENSCs were isolated from dissected intestines of WT Sprague Dawley rat pups (P7) via FACS using antibodies raised against p75 (Fig. 2a shows a typical FACS profile). After 1 week in culture, p75+ cultures were harvested and analysed by qRT-PCR. This revealed expression of the neural crest progenitor marker *Sox10*, as well as the pan-neuronal marker *Tuj1* and the glial marker *S100b* (Fig. 2b). Mature neuronal markers were also detected at low levels, including *Gls1, Nos1, Tph1, Chat* and *Gad1* (indicating the production of the neurotransmitters glutamate, nitric oxide, serotonin, acetylcholine and gamma-aminobutyric acid, respectively). Brightfield analysis of 1 week-old p75+ cultures revealed numerous cells with neuronal-like morphology (Fig. 2c), with neurospheres typically forming after approximately 2 weeks (Fig. 2d). Immunofluorescent investigation revealed several Ki67+ dividing cells (Fig. 2e), indicating that a subpopulation of cells likely maintained a ‘stem cell’ state to some degree. However, the majority of cells were TuJ1+ (Fig. 2f), with some differentiation towards specific neuronal subtypes including nNOS+ (Fig. 2g) and 5HT+ (Fig. 2h) neurons. This diversity was typical of the population of ENSC-derived cells utilised for transplantation, and henceforth referred to as ENSCs.

### Transplanted ENSCs survived transplantation, and LV-ChABC injection resulted in breakdown of CSPGs

Having determined that ENSCs are capable of differentiation into a variety of neuronal subtypes relevant for replenishing cells lost through SCI, we next assessed their ability to survive in the spinal cord injury zone, with and without the addition of ChABC-containing lentivirus. 3 days following induction of a spinal cord contusion, animals assigned to the treatment groups received ENSC transplantation and/or LV-ChABC injection. Spinal cords and other tissues were harvested 16 weeks post-injury.

**Fig. 3:**
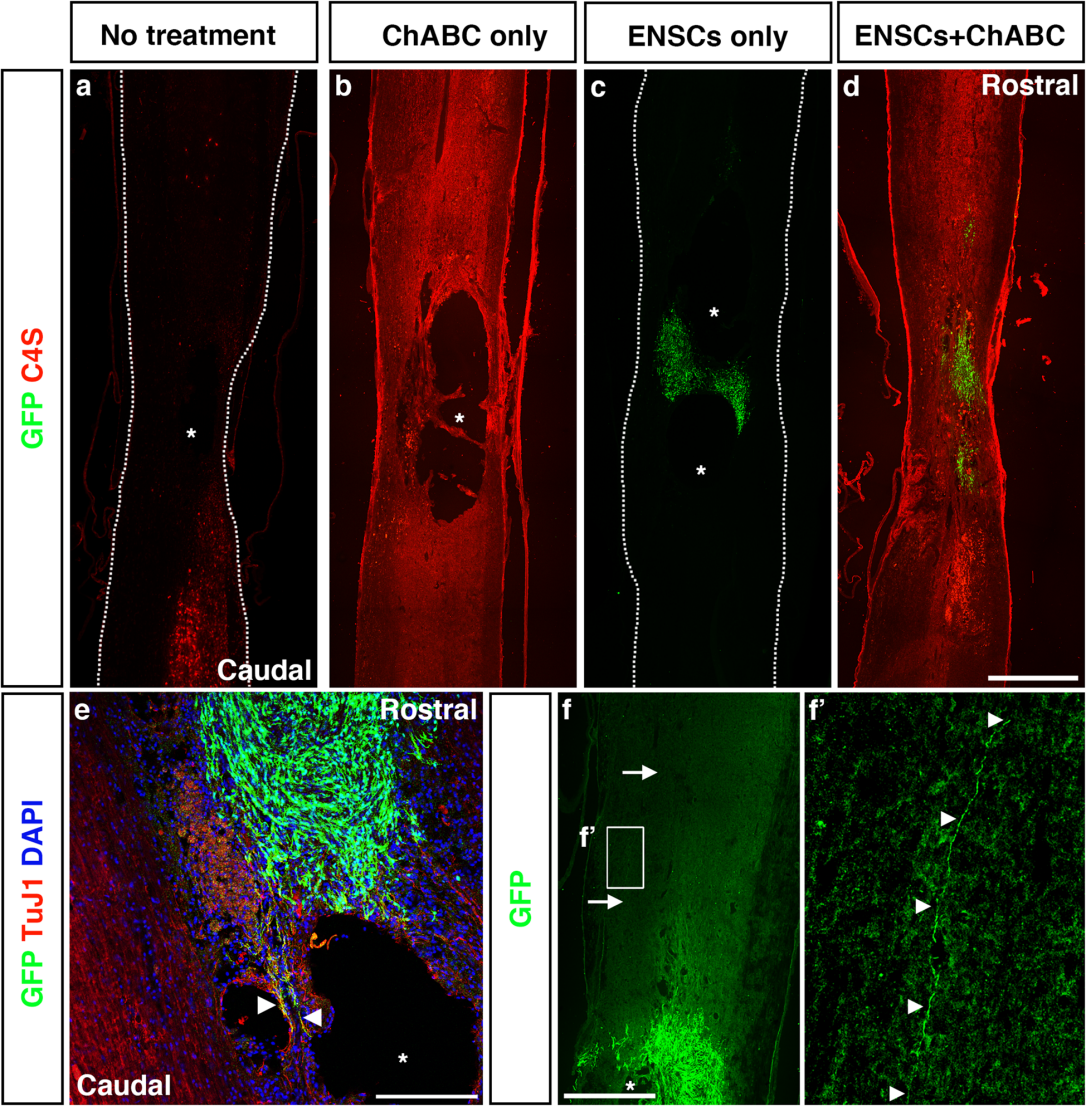
Immunoflourescent staining reveals the presence of CSPG breakdown products and ENSCs 16 weeks post-transplantation. Longitudinal cryosections of the spinal cords harvested at 16 weeks post-injury were analysed for markers indicative of successful treatment. **a**–**d** Representative images of the spinal cords in the untreated (**a**), ChABC-treated (**b**), ENSC-treated (**c**) and ENSC+ChABC-treated (**d**) groups. Asterisks indicate lesion cavity. Chondroitin-4-sulfate disaccharides (C4S), a breakdown product of CSPGs, were detected by immunoflourescent staining following treatment with ChABC (**b**, **d**). In rats receiving ENSC transplantation, ENSCs could be seen within the spinal cord injury zone (**c**, **d**). ENSCs extended substantial processes from the transplantation site (**e**, arrowheads), often for several millimetres (**f**, arrows indicate maximum detected length of a single GFP+ fibre). The boxed area in **f** is shown at a higher magnification in (**f**’). Arrowheads indicate the path of the GFP+ fibre. Scale bar—**d** 1 mm, **e** 200 μm, and **f** 500 μm

We first assessed CSPG digestion by ChABC (via detection of Chondroitin-4-sulfate disaccharides (C4S), a product of ChABC-mediated degradation of CSPGs) and ENSC survival (via immunostaining with an anti-GFP antibody). In both the ENSC+ChABC and ChABC-only groups, C4S+ staining was visible throughout much of the thoracic spinal cord, appearing concentrated around the lesion cavity (Fig. 3b, d). The untreated and ENSC-only groups showed no specific staining (Fig. 3a, c), although background autofluorescence (most likely macrophages) was present in several sections (Fig. 3a).

Numerous GFP+ ENSCs were found within the spinal cord in both ENSC-only and ENSC+ChABC-treated animals (Fig. 3c, d). These were mostly located at the rostral edge of the lesion, though substantial spread towards the caudal limit of the lesion through dorsal or ventral spared tissue was frequently observed (in some animals, simultaneous spread both ventral and dorsal to the lesion was noted). Transplanted ENSCs were almost invariably found in one or more large clusters and very rarely found as solitary cells. Both cell bodies and processes of transplanted ENSCs appeared to align along the A/P axis of endogenous tracts. In two animals, transplanted ENSCs crossed the injury zone through a dorsal-ventral ‘bridge’ which bisected the injury zone (Fig. 3c). In samples treated with both ENSCs and ChABC, transplanted cells frequently co-localised with areas of C4S+ staining. Transplanted ENSCs extended extensive TuJ1+ processes into surrounding host tissue (Fig. 3e). These projected both towards neighbouring ENSCs and into the surrounding SC tissue (Fig. 3e, arrowheads). In several instances, single axons projected rostrally from the transplant site for several millimetres (Fig. 3f, arrows, and f’, arrowheads).

### Transplanted ENSC spread and survival was not affected by co-application of ChABC

**Fig. 4:**
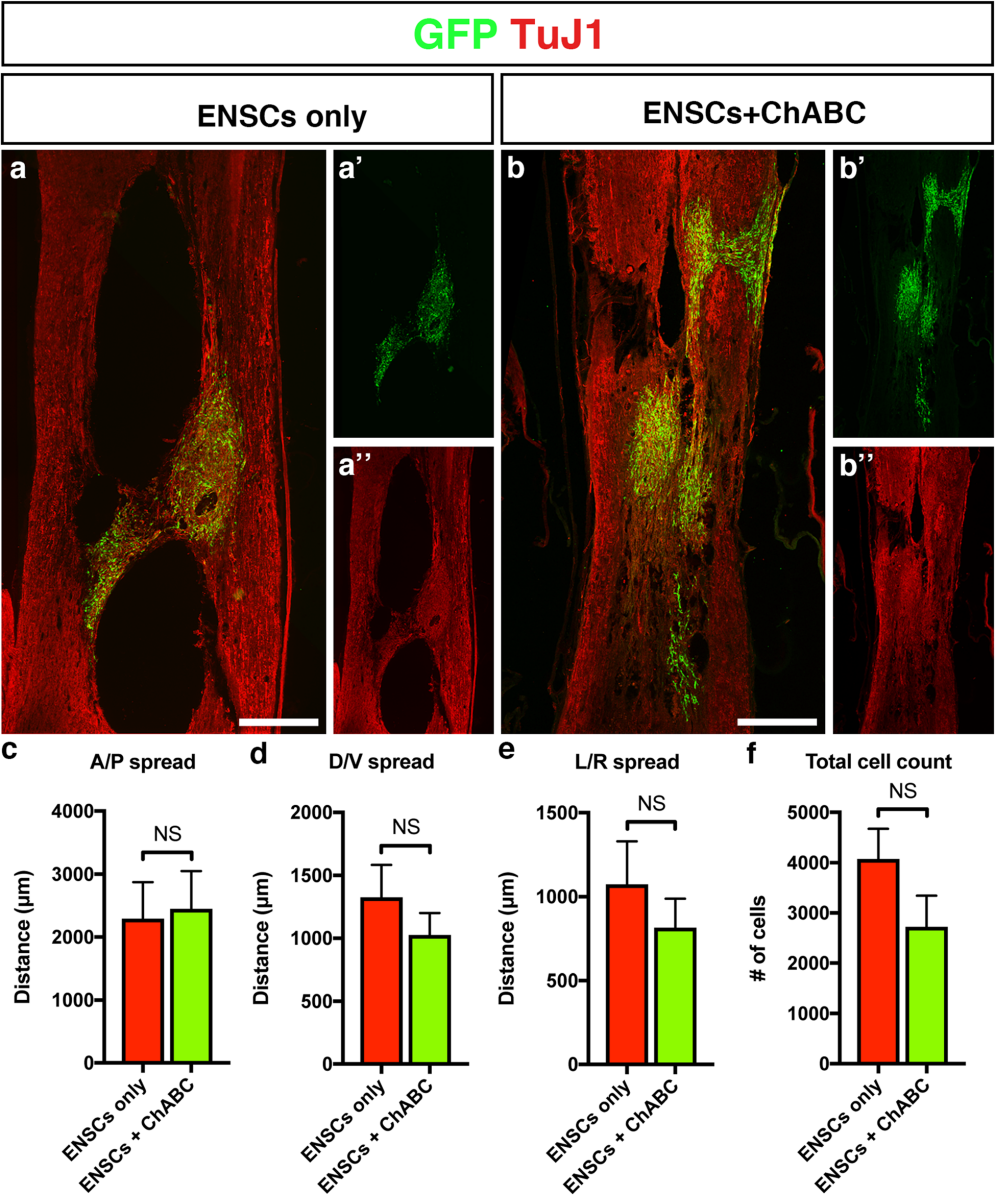
Application of ChABC has no effect on ENSC spread/behaviour. **a**, **b** Representative images of transplanted ENSCs within the ENSC-treated (**a**) and ENSC+ChABC-treated (**b**) spinal cords. At the time of tissue harvest (16 weeks post-injury), numerous GFP+ cells were found within sagittal spinal cord sections of both the ENSC-only group (**a**) and the ENSC+ChABC groups (**b**). **c**–**f** Serial spinal cord sections spanning the extent of GFP+ cell detection were analysed for cell spread across the anterior/posterior (**c**), dorsal/ventral (**d**) and left/right planes (**e**), as well as for cell survival (**f**). No significant differences were detected in any parameter between animals treated with ENSCs+ChABC or with ENSC-only. Data are represented as mean ± s.e.m. Scale bars—**a**, **b** 500 μm

To determine if there was any effect of ChABC on ENSC behaviour post-transplantation, serial spinal cord sections of rats receiving either ENSC-only (Fig. 4a) or ENSCs+ChABC (Fig. 4b) were stained with an anti-GFP antibody and imaged. No significant difference in cell spread between the ENSC-only and ENSCs+ChABC-treated animals was detected, across either the anterior/posterior (A/P, 2294.15 ± 581.2 μm vs 2451.05 ± 601.2 μm, *p* = 0.8573, Fig. 4c), dorsal/ventral (D/V, 1325 ± 259.4 μm vs 1025 ± 175 μm, *p* = 0.3747, Fig. 4d) or left/right (L/R, 1074.55 ± 255.6 μm vs 815.45 ± 173.2 μm, *p* = 0.4335, Fig. 4e) planes. The numbers of transplanted GFP+ ENSCs detected in the two groups also was not significantly different (4077.75 ± 598.7 μm vs 2724.5 ± 618.5 μm, *p* = 0.167, Fig. 4f). Similarly, quantification of the density of GFP+ pixels revealed no significant difference between rats receiving ENSC-only and ENSCs+ChABC (542,499.22 ± 180,444 vs 543,940.69 ± 170,462, *p* = 0.9956, data not shown). By all parameters quantified, co-treatment with ChABC had no effect on transplanted ENSC survival or spread.

### Combined ENSC and ChABC treatment significantly improved lesion pathology

**Fig. 5:**
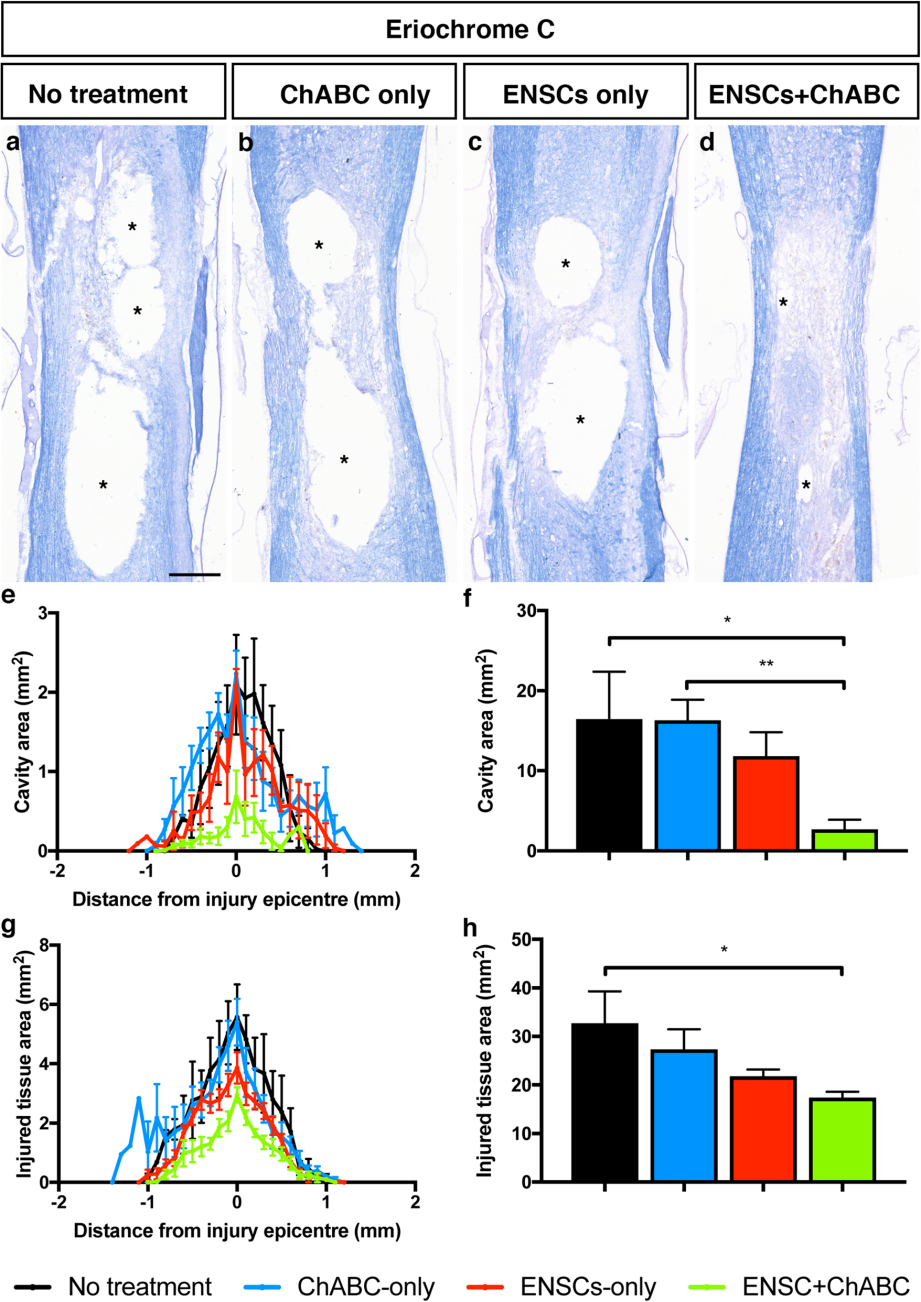
Combined treatment of SCI with ENSCs and ChABC resulted in significant reductions in tissue pathology, as assessed by eriochrome cyanine staining. **a**–**d** Representative sagittal sections of the spinal cords from each treatment group harvested from animals sacrificed 16 weeks post-injury and stained for eriochrome C. **e**–**h** Summary data of cavity area (**e**, **f**) or injured tissue area (**g**, **h**) analysis. ENSC transplantation or ChABC application applied as single treatments did not significantly affect the cavity size or the area of injured tissue. However, in the combined treatment group, there was a significant decrease in both cavity area and the area of injured tissue compared to the non-treated group. Data are represented as mean ± s.e.m. *Indicates significance (*p* ≤ 0.05). Scale bar—**a** 500 μm

To assess whether stem cell transplantation and/or ChABC therapy had any effect on lesion pathology following injury, serial sections were stained with eriochrome C (Fig. 5a–d). Quantification of the cavity area revealed differences between groups (Fig. 5e), and this was confirmed as significant with ANOVA analysis (Fig. 5f, *F*(3, 16) = 5.73, *p* = 0.0074). There were no significant differences in the average total cavity size compared to the untreated group in rats treated with ChABC alone (16.29 ± 2.563 vs 16.47 ± 5.919 mm^2^, *p* = > 0.999) or stem cells alone (11.85 ± 2.968 vs 16.47 ± 5.919 mm^2^, *p* = 0.7453). However, cavity area was significantly reduced in the ENSC+ChABC combination treatment group compared to non-treated animals (2.72 ± 1.183 vs 16.47 ± 5.919 mm^2^, *p* = 0.0309, Fig. 5f).

Lesion cavities were surrounded by injured tissue, notable in both disrupted organisation and poor eriochrome C staining. To determine whether the reduced cavity area observed in the ENSC+ChABC group corresponded to an expanded area of injured tissue, the lesioned tissue area was also quantified (Fig. 5g). Quantification of the area of injured tissue yielded results closely mirroring that of the cavity area (Fig. 5h). ANOVA analysis confirmed a significant difference between groups (*F*(3, 16) = 3.622, *p* = 0.0362). Although there was a trend for a decrease in lesioned tissue area in the single treatment groups these were not significantly different for either ChABC treatment (27.35 ± 4.127 vs 32.72 ± 6.582 mm^2^, *p* = 0.728) or ENSC transplantation (21.83 ± 1.353 vs 32.72 ± 6.582 mm^2^, *p* = 0.213) compared to the untreated group. Again, only the combined treatment resulted in a statistically significant decrease in injured tissue area (17.43 ± 1.195 vs 32.72 ± 6.582 mm^2,^ *p* = 0.0408). Taken together, these results indicate that the combined treatment results in an increase in the area of preserved tissue, by decreasing both the injury cavity and the area of injured tissue.

### Transplanted ENSCs project axons through and across the injury zone and result in a significant increase in the number of retrograde-labelled neurons crossing the injury site

**Fig. 6:**
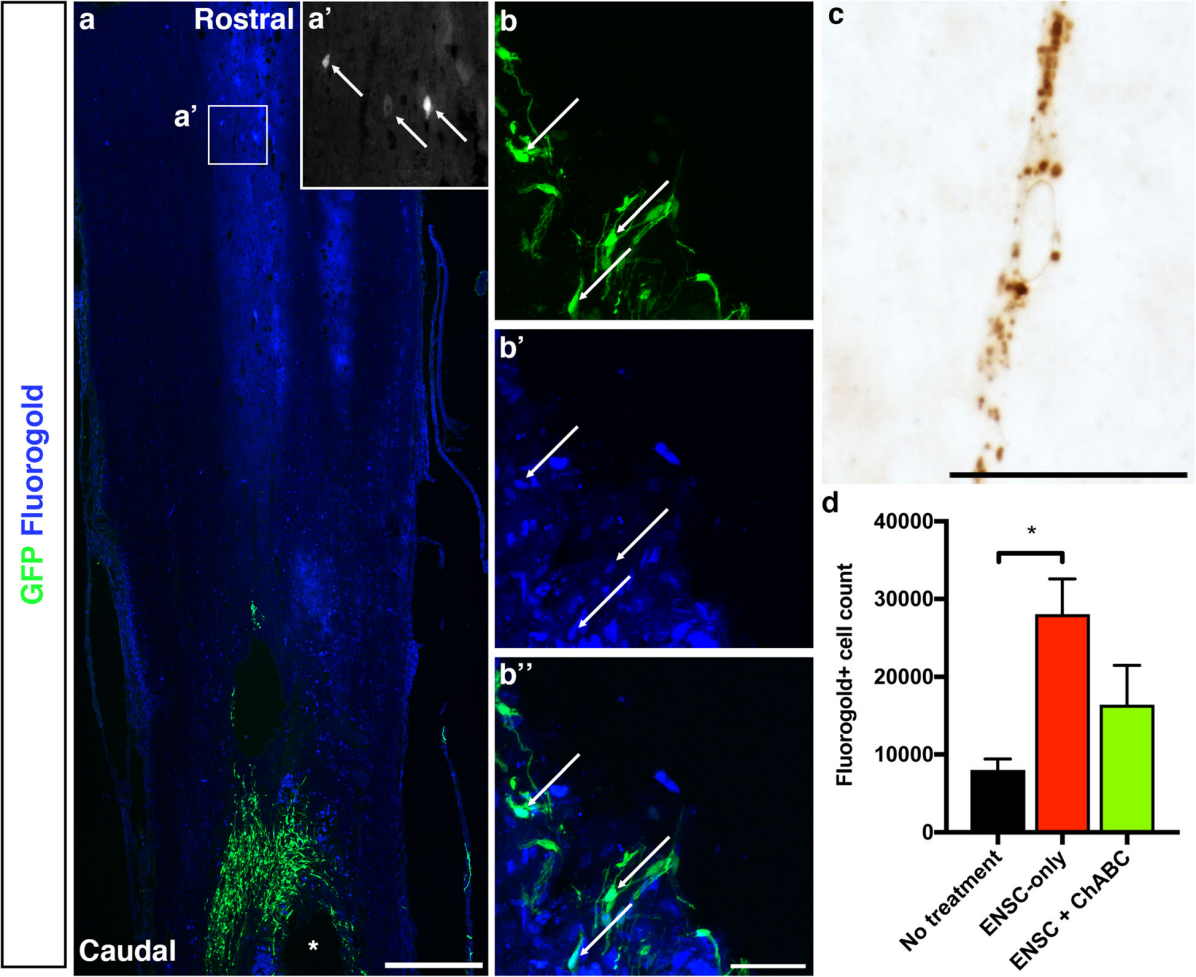
Transplanted ENSCs project axons through and past the injury site to reach caudal spinal cord regions. Fluorogold, a retrograde tracer, was injected caudal to the injury site (T12) at 15 weeks post-injury (1 week prior to sacrifice of animals at week 16). **a** Representative tile scan of the injury epicentre and rostral spinal cord of a rat treated with ENSCs only. Asterisk indicates injury cavity. **a’** High magnification of boxed area in (**a**), indicating FG-labelled neurons (arrows). Frequent instances of transplanted, GFP+ ENSCs (**b**) co-labelled with FG (**b**’) were found (**b**” shows merged image of **b** and **b**’). Following incubation with a fluorogold antibody and development with diaminobenzidine (**c**), the number of FG+ cells rostral to the lesion was quantified using unbiased stereology (**d**). Data are represented as mean ± s.e.m. *Indicates significance (*p* ≤ 0.05). Scale bars: **a** 500 μm, **b** 50 μm, and **c** 25 μm

To determine whether transplanted ENSCs were capable of long distance axonal projection bypassing the lesion, the transplanted rat spinal cords were injected with a retrograde tracer, fluorogold (FG) caudal to the injury site (T12), resulting in a substantial number of FG-labelled neurons rostral of the lesion (Fig. 6a). The morphology of the stained cells was variable in terms of both size and number of processes (Fig. 6a’). We also found multiple instances of FG+ transplanted ENSCs within the transplant site, indicating that these cells were sending projections past the injury site to distant caudal regions (Fig. 6b, arrows indicate co-labelled cells). To determine whether the observed tissue sparing resulted in an increased number of tracts bypassing the lesion, tissue sections were incubated with antibodies raised against fluorogold and developed with diaminobenzidine (Fig. 6c) and the number of fluorogold+ neurons rostral to the lesion was quantified in the untreated, ENSC-only and ENSC+ChABC groups. No significant difference was observed between the ENSC+ChABC and ENSC-only groups (16,421.3 ± 5062 vs 28,046.7 ± 4547, *p* = 0.5158, Fig. 6d). However, a significant difference was observed between the ENSC-transplanted and untreated groups (28,046.7 ± 4547 vs 8057.2 ± 1349, *p* = 0.0287, Fig. 6d).

### Transplanted ENSCs do not engraft in peripheral organs or form tumours

Undesired migration and tumorigenesis of transplanted cells is a potential risk of stem cell-based treatments. Upon gross dissection and careful examination, no tumours were observed in any of the transplanted animals after 16 weeks, and mortality within treatment groups was not found to be different compared to non-transplanted animals over the course of the study. To test for the presence of transplanted cells in ‘off target’ locations, we used PCR to detect the expression of *Gfp*, indicative of the presence of transplanted ENSCs in peripheral organ tissue samples. The lung, liver, kidney, and spleen were assessed alongside cryosections of transplanted SC tissue, which had been confirmed to contain GFP-expressing cells and was therefore used as a positive control. While all samples revealed expression of *Gapdh*, only positive control samples of transplanted SC revealed expression of *Gfp* (Supp. Figure 2, arrow), with no *Gfp* expression detected in any of the other organs examined.

## Discussion

Here, we demonstrate the therapeutic potential of a combined treatment of ENSCs and ChABC for SCI. ENSCs transplanted into the injured spinal cord of immunocompetent rats survived and formed continuous bridges across the injury zone. The combined treatment resulted in significant reductions in the cavity area of injured spinal cords, and transplanted cells projected axons across the injury site and increased the number of cells projecting axons caudally through the injury site. At the latest time point examined, the combined treatment of ENSCs+ChABC led to a small but significant increase in the ability of injured rats to traverse the horizontal ladder. These data suggest that ENSCs, particularly in combination with other therapeutic interventions, may serve as a viable substitute for other, less widely available stem cell sources.

The horizontal ladder test has been utilised previously as an assessment of motor function recovery following SCI [43]. In the current study, we found no significant difference between the groups until the final week assessed, week 16, at which point the combined treatment group performed significantly better than the no treatment and ENSC-only treatment. These results are in line with previous publications examining the combinatorial application of neural stem cells and ChABC [46, 47], which have reported significant benefits in a wide variety of behavioural tests, including a panel of gait analyses and Basso, Beattie and Bresnahan (BBB) analysis. The modest improvements observed in the current study are promising and should be further evaluated with a larger sample size and more comprehensive variety of behavioural tests to ascertain the true extent of functional recovery.

Simultaneous treatment with ChABC and ENSC transplantation resulted in significant reductions in both affected tissue area and cavity area, both of which are often used as a neuroprotective marker in cell transplant studies [50, 56–58]. ChABC+ENSC treatment caused a decrease in cavity area of approximately 83.5%. Comparatively, Führmann et al., who treated injured spinal cords with ChABC immediately following injury and transplanted cells 1 week later, noted reductions in cavity volume of around 50% following application of induced pluripotent stem cell (iPSC)-derived neuroepithelial cells, either alone or in combination with ChABC [59]. Nori et al., who administered ChABC 6 weeks following injury and transplanted cells 1 week later, detected no such improvement in tissue preservation (although they noted impressive locomotor recovery) following combined treatment of human oligodendrogenic neural progenitor cells with ChABC [60]. Combined, these findings suggest that for stem cell-based treatments, early interventions in the acute injury phase may be required to limit and/or reverse cavity formation. The effect of timing is also likely a key factor in mediating neuroprotective effects of ChABC-based interventions [61]. Previous publications have shown that ChABC treatment can have beneficial immunomodulatory effects when administered immediately post-injury via lentiviral delivery (Bartus et al., 2014; James et al., 2015) or when given as high-dose bolus injections of enzyme either prior to or within 1 h post-injury [62]. In contrast to previous studies, we observed no significant difference in the cavity area between the ChABC-only and untreated groups. This is likely due to differences in timing, since we observed a similar pattern of widespread CSPG digestion to that of Bartus et al. [50], but LV-ChABC was administered with a 3-day delay. Importantly, Bartus et al. showed that by 3 days post-injury LV-ChABC treatment had significantly altered the immune cell phenotype. Thus, the 3-day delay in treatment likely underlies the lack of neuroprotection due to a lack of early immunomodulation. Notably, a publication by Sarveazad et al. noted the beneficial effects of ChABC on cavity size (alone or in combination with human adipose-derived stem cells) when transplanted 1 week post-injury [63]. This treatment involved direct injection of the enzyme rather than a viral construct, making a direct comparison difficult. However, the current study used injections of ChABC lentivirus at a concentration of 1 × 10^9^gc—a slightly lower titre compared to our previous publication [64]. Sarveazad et al. used a particularly high dose of ChABC (10 μL of 100 U/mL) compared with other publications [46, 47, 65]. Previous publications have demonstrated that the concentration of ChABC administered to the injured spinal cord is vital for adequate digestion of CSPGs [65], and this discrepancy may explain the variation in results. Interestingly, while acute administration of LV-ChABC may be crucial for mediating immunomodulatory and neuroprotective effects when delivered alone, in the present study, we found that in combination with ENSCs, LV-ChABC enables significantly reduced injury pathology over ENSCs alone.

As co-application of ChABC with ENSC transplantation enhanced cavity reduction compared to ENSCs alone, it may have been expected that ChABC was fulfilling a supporting role towards transplanted ENSCs. Surprisingly however, we found no significant difference in transplanted cell characteristics between the ENSC-only and ENSCs+ChABC groups, as assessed by transplanted cell spread analysis, GFP+ transplanted cell counting and GFP+ immunostaining threshold analysis of transplanted tissue. These results are in line with a previous publication which found no difference in survival of cells when transplanted with or without ChABC-loaded microtubes [66]. The lack of a difference in any of the parameters examined in the current study suggests that the two treatments have independent, supportive effects on endogenous tissue. ChABC has been shown to enhance both endogenous axon regeneration and preservation of vulnerable neuronal tracts after SCI ([43], reviewed in [67]), and additional studies have demonstrated that transplanted stem cells can secrete neurotrophic factors to support the survival of host tissue [68, 69]. ENSCs transplanted into the injured SC in the current study may exert a similar paracrine-driven effect on endogenous tissue. Although to the best of our knowledge, it is currently unknown what, if any, factors may be secreted by ENSCs, in their paper utilising transplantation of ENSCs into the injured mammalian brain, Belkind-Gerson et al. noted that the transplanted cells appeared to stimulate endogenous neurogenesis, possibly by secretion of stimulatory factors [35]. In addition, numerous studies have demonstrated the potential of transplanted cells, such as olfactory ensheathing glia (OEGs), to form bridges for endogenous axons to follow across the injury site [70, 71]. It is possible that ENSCs fulfilled a similar role in the current study, with ChABC supporting the sprouting or survival of endogenous axons across these bridges. Indeed, a previous publication by Carwardine et al. noted increased endogenous sprouting in a cervical dorsal crush spinal cord lesion following transplantation of ChABC-expressing olfactory ensheathing cells (OECs) [72]. In this study, the authors noted significantly more endogenous sprouting in the animals transplanted with OECs+ChABC, compared to OECs alone, again indicating a synergistic effect of the two treatments. In the current study, we found frequent instances of transplanted ENSCs closely associated with TuJ1+ endogenous tracts, likely helping to preserve surviving and/or sprouting tracts.

Transplanted ENSCs often extended in a continuous chain across the injury site, potentially providing a bridge for regenerating endogenous axons to bypass the lesion. Maximum spread or outgrowth of transplanted ENSCs reached 4.2 mm along the A/P axis. Using a similar methodology, we have previously shown a spread of approximately 5.5 mm^2^ following transplantation of ENSCs into the colon, arguably their ‘natural’ environment [34]. The similar level of spread suggests that ENSCs are able to adapt to the CNS environment. While the current study marks the first time ENSCs have been transplanted into the mammalian spinal cord following injury, publications describing the spread of other stem cells post-transplant are numerous and appear to show high variation. For example, Lepore and Fisher noted cell migration of up to 15 mm following transplantation of embryonic neuronal and glial-restricted progenitors embedded in a collagen matrix [73]. However, the authors noted that the degree of spread was highly inconsistent between animals. Other publications have instead measured the distance of cell projections, often using co-application of additional factors to boost outgrowth. Wictorin and Bjorklund observed fibre outgrowth up to 10 mm from the transplant site [74]. Lu et al. observed extension of axons up to ~ 20 mm [75] following the combinatorial application of fibrin scaffolds supplemented with a cocktail of growth factors including BDNF and GDNF. Future work will aim to optimise conditions to promote the outgrowth of transplanted ENSC processes.

A major obstacle for promoting motor/sensory recovery following SCI is the difficulty in encouraging axonal sprouting of endogenous or transplanted neurons (reviewed in [76]). This has led to several groups investigating combined therapies, such as co-application of stem cells with scaffolds, in an attempt to encourage stem cell-derived axon growth [77]. As such, the frequent instances of FG+ ENSCs found around the injury zone in both the ENSC-only *and* ENSC+ChABC groups in the current study are very encouraging and indicate that a subpopulation of transplanted cells project axons through/past the injury site to reach caudal host tissue. FG is a retrograde tracer and is not known to diffuse from labelled cells. Therefore, it is incredibly unlikely that the FG could have been passed from a labelled, endogenous cell to a transplanted GFP+ cell. Following the quantification of the total number of FG+ cells rostral to the lesion, we found significantly higher numbers of FG+ neurons in the ENSC-transplanted group compared to the non-treated group. Similar increases in the number of cells crossing the lesion have been noted following transplantation of cells from established stem cell sources. Zhou et al. observed an increase in the number of FG+ axons crossing the injury site following combined treatment with bone marrow-derived mesenchymal stem cells and propofol (an anaesthetic thought to be neuroprotective, possibly via its antioxidant and apoptosis-inhibiting properties) [78]. Other studies utilising neural crest-derived cells such as Schwann cell or OEG cell grafts have also resulted in increased FG+ neurons crossing the injury site [79]. This further demonstrates that, at least for the purposes of neuroprotection, ENSCs can serve as a viable substitute for other, more difficult to access stem cell sources.

As well as the effect of transplanted cells upon endogenous tissue, the fate of the transplanted cells themselves is of key importance. Previous reports of cell-based therapies have documented such extensive glial differentiation following transplantation into the spinal cord as to suggest that astrocytic differentiation of transplanted progenitor cells may be the default pathway [80, 81]. In contrast, our in vitro qRT-PCR data revealed expression of a panel of mature neuronal markers, including *Gls1, Nos1, Tph1, Chat* and *Gad1*, similar to previous work by our lab on chick-derived ENSCs [38]. We used immunofluorescence to confirm the presence of 5HT+ and nNOS+ neurons. Thus, transplanted ENSCs have the potential to replace neurons lost through the injury process, and indeed, we found frequent TuJ1+ ENSCs post-transplantation. It must be noted that we also detected expression of the glial marker S100b in our in vitro studies. Further work will determine the specific neuronal subtypes ENSCs differentiate into post-transplantation, and whether they contribute glia to the injury zone.

Tumorigenesis following stem cell transplantation is a recognised concern of stem cell applications [39, 40, 82–84]. In a recent publication by Hirota et al., teratomas were observed in nearly one third of mice transplanted with iPSC-derived cells 9–10 weeks post-transplantation [85], highlighting the need for thorough safety assessments before progression to clinical trials. Encouragingly, we found no evidence of tumour formation in rats at 16 weeks post-transplant. We also used PCR analysis of peripheral organs to detect the spread of GFP-labelled transplanted cells, but only found GFP expression in transplanted SC tissue, in line with long-term safety results previously published by our group [86]. The results described in the current study therefore provide initial evidence of the safety of ENSC transplantation into the spinal cord, but further long-term analysis of ENSC proliferation post-transplantation, and genetic sequencing to confirm that genetic instability does not occur in ENSCs (as it can in iPSCs [87]) will be required.

Notably, it is likely that any harvest and expansion of ENSCs will take longer than 3 days, and so, transplantation at 3 days post-injury, as in the current manuscript, will likely be difficult to replicate in the clinical setting. Future experiments will aim to determine the earliest time point at which patient-derived ENSCs could be expanded sufficiently for transplantation. Similar to previous reports [35, 88, 89], in our study, ENSCs were expanded in vitro for 2 weeks prior to transplantation. Encouragingly, work by Didangelos et al. demonstrated that ChABC applied 1 h post-injury and hence every 48 h (up to 7 days) was able to promote an anti-inflammatory environment within the injury zone [62]. A comprehensive evaluation of the potential of therapies such as ChABC application to prime the injury environment while autologous stem cells are expanded in vitro will be vital.

## Conclusions

We show that ENSCs isolated from the ENS of the GI tract have therapeutic potential for the treatment of SCI, in terms of reducing cavity/injured tissue area and projecting axons rostrally through the injury zone to reach spared tissue caudal to the lesion. These effects were enhanced when combined with ChABC and led to a modest but significant functional improvement as assessed by the horizontal ladder test. Transplanted ENSCs survived up to the latest time point examined (16 weeks), and preliminary evidence suggests that ENSCs did not migrate to undesired areas. These results strongly encourage further exploration of the use of ENSCs for both SCI and a range of CNS disorders.

## Supplementary Information

**Additional file 1: Supp. Figure 1.** Chondroitinase lentiviral vector construct. A lentivirus containing the chondroitinase plasmid and the PGK promoter were used to drive chondroitinase expression following injection into the injured spinal cord.

**Additional file 2: Supp. Figure 2.** ENSCs do not spread to peripheral organs, as assessed by PCR detection of GFP. Samples of peripheral organs, including the lung, liver, kidney and spleen were harvested from animals that had received transplantations of ENSCs into the SC. Genomic DNA was extracted and primers for *Gapdh* (control) and *Gfp* were used to assess the presence of transplanted cells. Genomic DNA extracted from cryosections of transplanted rat SC (TP SC) confirmed to have GFP+ ENSCs was used as a positive control, and sterile H_2_O was used as negative control. *Gfp* was only detected in the transplanted SC confirmed to contain GFP+ cells (arrow).

## Data Availability

The datasets used and/or analysed during the current study are available from the corresponding author on reasonable request.

## Abbreviations

A/P: Anterior/posterior
ChABC: Chondroitinase ABC
ChAT: Choline acetyltransferase
CSPG: Chondroitin sulfate proteoglycans
CNS: Central nervous system
C4S: Chondroitin-4-sulfate disaccharides
D/V: Dorsal/ventral
EC: Eriochrome C
eGFP: Enhanced green fluorescent protein
ELISA: Enzyme-linked immunosorbent assay
ENS: Enteric nervous system
ENSC: Enteric neural stem cell
FG: Fluorogold
GAD: Glutamic acid decarboxylase
Gapdh: Glyceraldehyde 3-phosphate dehydrogenase
GFP: Green fluorescent protein
GI: Gastrointestinal
Gls1: Glutaminase 1
i.p.: Intraperitoneal
iPSC: Induced pluripotent stem cell
L/R: Left/right
LV: Lentivirus
nNOS: Neuronal nitric oxide synthase
OEGs: Olfactory ensheathing glia
SCI: Spinal cord injury
s.e.m.: Standard error of the mean
SIN: Self-inactivating
Sox10: SRY-Box transcription factor 10
Tph1: Tryptophan hydroxylase 1
TuJ1: Beta tubulin III
WPRE: Woodchuck Posttranscriptional Regulatory Element
5HT: Serotonin
