# Supplementary figures and images for: Combined treatment with enteric neural stem cells and chondroitinase ABC reduces spinal cord lesion pathology
Source: Stem Cell Res Ther. 2021 Jan 6;12:10. doi: 10.1186/s13287-020-02031-9 (PMC7789480; doi:10.1186/s13287-020-02031-9)

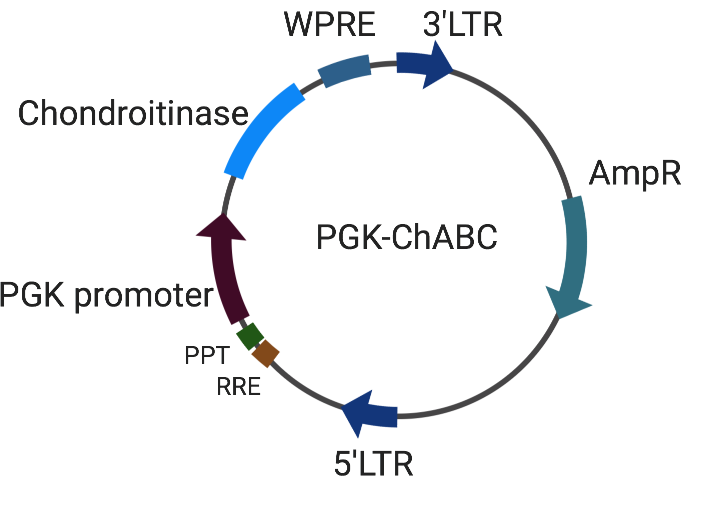

Supplement: Supplementary file 1 — Additional file 1: Supp. Figure 1. Chondroitinase lentiviral vector construct. A lentivirus containing the chondroitinase plasmid and the PGK promoter were used to drive chondroitinase expression following injection into the injured spinal cord. [file 13287_2020_2031_MOESM1_ESM.tif]

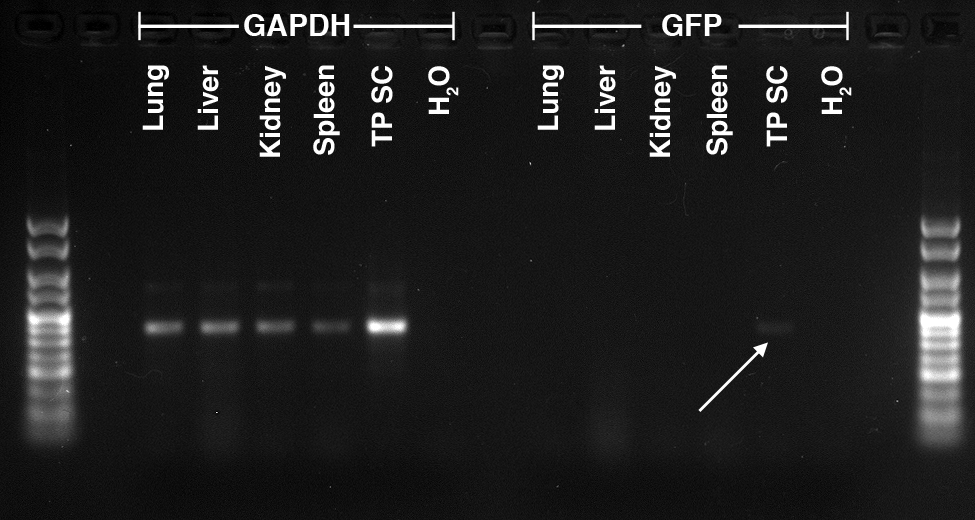

Supplement: Supplementary file 2 — Additional file 2: Supp. Figure 2. ENSCs do not spread to peripheral organs, as assessed by PCR detection of GFP. Samples of peripheral organs, including the lung, liver, kidney and spleen were harvested from animals that had received transplantations of ENSCs into the SC. Genomic DNA was extracted and primers for Gapdh (control) and Gfp were used to assess the presence of transplanted cells. Genomic DNA extracted from cryosections of transplanted rat SC (TP SC) confirmed to have GFP+ ENSCs was used as a positive control, and sterile H2O was used as negative control. Gfp was only detected in the transplanted SC confirmed to contain GFP+ cells (arrow). [file 13287_2020_2031_MOESM2_ESM.tif]
